# Multimodal Opioid-Sparing Analgesia for Lumbar Decompression in a Patient With Opioid-Associated Sphincter of Oddi Symptoms: A Case Report

**DOI:** 10.7759/cureus.116500

**Published:** 2026-09-18

**Authors:** Asad Shabbir Cheema

**Affiliations:** 1 Anaesthesia, University Hospital Limerick, Limerick, IRL

**Keywords:** acute postoperative pain, lumbar spinal decompression, multimodal analgesia, opioid-sparing analgesia, sphincter of oddi dysfunction

## Abstract

Sphincter of Oddi symptoms can make perioperative analgesia challenging, particularly in patients who have previously developed severe biliary-type pain after opioid administration. We describe the perioperative management of a 51-year-old woman with a clinically diagnosed history of sphincter of Oddi dysfunction. During previous surgery, she reported developing severe right upper quadrant pain and nausea shortly after receiving opioids. She subsequently underwent approximately three hours of L4/5 lumbar decompression under general anaesthesia. An opioid-sparing multimodal analgesic regimen was used, including paracetamol, ibuprofen, tramadol, ketamine, intravenous lidocaine, clonidine, pregabalin, and local levobupivacaine infiltration. Fentanyl was given at induction, and remifentanil was used during the procedure. Postoperatively, regular tramadol was continued with pregabalin, clonidine, and a lidocaine patch. At 24 hours, pain was 4/10 at rest and increased to 8/10 during physiotherapy. By 48 hours, pain at rest was 4/10. She did not develop the right upper quadrant or biliary-type pain that she had previously experienced with opioids. She had nausea in the recovery area, which settled with standard antiemetic treatment. She mobilised successfully with physiotherapy and was satisfied with her pain management, stating that she would choose the same approach again. This case describes a practical opioid-sparing approach in a patient with a history of opioid-associated sphincter of Oddi symptoms. Although opioids could not be completely avoided, multimodal analgesia allowed their use to be limited while providing acceptable postoperative pain control.

## Introduction

Opioid-related effects on the sphincter of Oddi can make perioperative analgesia challenging in patients who have previously developed biliary-type pain after opioid administration. Morphine has been shown to increase sphincter of Oddi pressure and contractile activity in human manometry studies, while other opioids appear to have variable effects [1,2]. This has traditionally led to caution when using opioids in patients with suspected sphincter of Oddi dysfunction, although the available evidence is limited and the effect is not the same for all opioids [3,4].

Tramadol may be different from some conventional opioids in this regard. Human manometry studies have found no adverse increase in sphincter pressure with tramadol, and one study reported a reduction in basal pressure and contractile amplitude. These findings are based on relatively small studies and do not establish that tramadol is completely safe in patients with sphincter of Oddi dysfunction [2,3]. At the same time, opioids may still have a role in perioperative analgesia for patients undergoing major surgery, including spine surgery, where postoperative pain can be significant. An opioid-sparing approach may therefore be a more realistic option than complete opioid avoidance in some patients [5,6].

Multimodal analgesia allows opioid requirements to be reduced by combining drugs with different mechanisms of action. In spine surgery, this may include paracetamol, non-steroidal anti-inflammatory drugs, gabapentinoids, ketamine, intravenous lidocaine, and local anaesthetic techniques [5-8]. We describe the perioperative management of a patient with a clinically diagnosed history of sphincter of Oddi dysfunction who had previously developed severe right upper quadrant pain and nausea after opioid administration and subsequently underwent lumbar decompression using a multimodal opioid-sparing analgesic regimen.

## Case presentation

A 51-year-old woman with a history of clinically diagnosed sphincter of Oddi dysfunction was scheduled for elective L4/5 lumbar decompression for radiculopathy. Her medical history included fibromyalgia, Crohn’s disease, rheumatoid arthritis, asthma, migraine, and irritable bowel syndrome. She had previously undergone bowel surgery with ileostomy, hysterectomy, cholecystectomy, and appendicectomy. She was an ex-smoker and had a history of postoperative nausea and vomiting.

Her diagnosis of sphincter of Oddi dysfunction was based on her previous clinical history rather than formal manometry. She reported that during a previous bowel operation, she developed severe right upper quadrant pain and nausea within a few minutes of receiving opioids. She described the pain as severe and out of proportion to the expected postoperative pain. The records from that admission were not available. She did not report any previous episodes of documented pancreatitis, cholangitis, or jaundice related to these symptoms, and she had never undergone sphincter of Oddi manometry. Her documented opioid allergies or intolerances included morphine, codeine, oxycodone, and tapentadol. Before surgery, she was taking tramadol 50 mg three times daily for fibromyalgia.

She underwent L4/5 posterior decompression, including laminectomy, right medial facetectomy, lateral recess decompression, and excision of a right L4/5 juxta-articular synovial cyst. The procedure lasted approximately three hours, and the estimated blood loss was 100 mL. General anaesthesia was maintained with sevoflurane and remifentanil. Fentanyl 100 µg was given at induction. Remifentanil was prepared at 50 µg/mL and infused at 10 mL/h for approximately 2.5 hours, giving a total dose of approximately 1.25 mg. It was stopped approximately 10 minutes before emergence.

Because of her previous symptoms, the aim was to minimise opioid exposure while still providing adequate analgesia. A multimodal approach was therefore used. She received tramadol 100 mg, ketamine 80 mg, intravenous lidocaine 150 mg, clonidine 100 µg, paracetamol 1 g, and ibuprofen 400 mg intraoperatively. The surgical team infiltrated the wound with 20 mL of 0.25% levobupivacaine (50 mg). Apart from the fentanyl and remifentanil used as part of the anaesthetic, no other opioid was given. A further 40 mg of ketamine was administered in recovery, giving a total perioperative ketamine dose of 120 mg.

Postoperatively, tramadol was increased to 100 mg three times daily. Pregabalin was increased from 75 to 100 mg, and clonidine 50 µg twice daily was continued. A lidocaine patch was also used. Six hours after surgery, her pain was 4/10 at rest and 5/10 with movement, increasing to 8/10 during physiotherapy. At 24 hours, pain was again 4/10 at rest and 8/10 during physiotherapy. By 48 hours, her pain at rest was 4/10 and was localised to the lumbar operative site.

Importantly, she did not develop the severe right upper quadrant or biliary-type pain that she had previously experienced after opioids. These symptoms did not recur during the postoperative period despite exposure to fentanyl, remifentanil, and tramadol. She developed nausea shortly after arrival in recovery and was treated with ondansetron and cyclizine. The nausea resolved within approximately two hours, and there were no further significant gastrointestinal symptoms.

She mobilised approximately 12 hours after surgery and was able to participate in physiotherapy. She remained in hospital over the weekend and was discharged on Monday. She was satisfied with the analgesic approach and said that she would choose the same strategy again.

## Discussion

The main challenge in this case was providing adequate analgesia while limiting opioid exposure in a patient who had previously developed severe right upper quadrant pain and nausea shortly after receiving opioids during surgery. Her history was suggestive of opioid-associated sphincter of Oddi symptoms, although the diagnosis had never been confirmed with manometry. This is important when interpreting the case, as the diagnosis was based on her clinical history rather than objective testing.

Morphine has been shown to increase sphincter of Oddi pressure and contractile activity in human manometry studies [1,2]. However, different opioids do not appear to have identical effects. In studies involving human subjects, tramadol did not produce the increase in sphincter pressure seen with morphine, and one study found a reduction in basal pressure and contractile amplitude [2,3]. This was one reason tramadol was continued in our patient, particularly as she was already taking it regularly for fibromyalgia. These findings do not mean that tramadol can be considered completely safe in patients with sphincter of Oddi dysfunction, but they provided some reassurance when choosing an opioid-sparing strategy.

We were not able to avoid opioids completely. Fentanyl was given at induction, and remifentanil was used during maintenance of anaesthesia. The effect of remifentanil on the sphincter of Oddi is less clear. There has been a case report describing apparent remifentanil-associated sphincter spasm that resolved after the infusion was stopped [9]. In our patient, no biliary-type pain developed during or after anaesthesia.

The main strategy was therefore to reduce the need for additional opioids by using several other analgesic agents. Multimodal analgesia is commonly used in spine surgery and can combine drugs with different mechanisms to improve pain control and reduce opioid requirements [5,6]. In this case, paracetamol, ibuprofen, pregabalin, ketamine, clonidine, intravenous lidocaine, and local levobupivacaine were used alongside tramadol. This provided reasonable postoperative analgesia. Pain was 4/10 at rest at 24 hours, although it increased to 8/10 during physiotherapy, and was 4/10 at rest by 48 hours.

Ketamine was included because of its analgesic and opioid-sparing effects and was also considered appropriate given the patient's background of chronic pain. Studies of ketamine in spine surgery have shown reductions in postoperative pain and opioid consumption, although the degree of benefit varies between studies [7]. Intravenous lidocaine was another component of the multimodal approach. Its use requires attention to dosing and overall local anaesthetic exposure, particularly when another local anaesthetic is also used for wound infiltration [8].

The most interesting finding in this case was that the patient did not develop the severe right upper quadrant or biliary-type pain that she had previously associated with opioids. This remained the case despite receiving fentanyl, remifentanil, and tramadol. We cannot say from this case which drug, or combination of drugs, was responsible for this outcome. It is also possible that her previous symptoms were not solely related to sphincter spasm. However, the clinical course suggests that an opioid-sparing approach was a practical way of managing her analgesia without reproducing the symptoms that had concerned her previously.

There are important limitations. The diagnosis of sphincter of Oddi dysfunction was based on the patient's history and had not been confirmed by manometry. The records from her previous surgery were unavailable, so the earlier episodes could not be independently verified. There was also no objective measurement of sphincter pressure before or after the current anaesthetic. Because several analgesic drugs were used together, the effect of any individual drug cannot be determined. Finally, this was not an opioid-free anaesthetic, and the findings should not be taken as evidence that fentanyl, remifentanil, or tramadol are routinely safe in patients with confirmed sphincter of Oddi dysfunction.

The practical lesson from this case was that complete avoidance of opioids may not always be necessary. In a patient with a convincing history of opioid-associated biliary pain, reducing opioid exposure and using several non-opioid analgesic techniques may be a reasonable alternative, provided the analgesic plan is individualised and the limitations of the available evidence are recognised.

## Conclusions

This case shows that a multimodal, opioid-sparing approach can provide satisfactory postoperative analgesia in a patient with a history of opioid-associated biliary pain. Although fentanyl, remifentanil, and tramadol were used, the patient did not develop the severe right upper quadrant pain that she had experienced with opioids during previous surgery. This does not establish that these drugs are safe in patients with sphincter of Oddi dysfunction, particularly as the diagnosis in this patient was clinical and was not confirmed with manometry. However, reducing opioid exposure and using a combination of non-opioid analgesic techniques provided a practical approach to her perioperative pain management. Further research is needed to clarify whether individual opioids differ in their effects on the sphincter of Oddi.
